# *ABCC9* Is Downregulated and Prone to Microsatellite Instability on ABCC9tetra in Canine Breast Cancer

**DOI:** 10.3389/fvets.2021.819293

**Published:** 2022-01-07

**Authors:** Pan Hao, Kai-yue Song, Si-qi Wang, Xiao-jun Huang, Da-wei Yao, De-ji Yang

**Affiliations:** College of Veterinary Medicine, Nanjing Agricultural University, Nanjing, China

**Keywords:** microsatellite instability, canine breast cancer, oncogenesis, the adenosine triphosphate binding cassette subfamily C member 9, loss of heterozygosity

## Abstract

Tumorigenesis is associated with metabolic abnormalities and genomic instability. Microsatellite mutations, including microsatellite instability (MSI) and loss of heterozygosity (LOH), are associated with the functional impairment of some tumor-related genes. To investigate the role of MSI and LOH in sporadic breast tumors in canines, 22 tumors DNA samples and their adjacent normal tissues were evaluated using polyacrylamide gel electrophoresis and silver staining for 58 microsatellites. Quantitative real-time polymerase chain reaction, promoter methylation analysis and immunohistochemical staining were used to quantify gene expression. The results revealed that a total of 14 tumors (6 benign tumors and 8 breast cancers) exhibited instability as MSI-Low tumors. Most of the microsatellite loci possessed a single occurrence of mutations. The maximum number of MSI mutations on loci was observed in tumors with a lower degree of differentiation. Among the unstable markers, FH2060 (4/22), ABCC9tetra (4/22) and SCN11A (6/22) were high-frequency mutation sites, whereas FH2060 was a high-frequency LOH site (4/22). The ABCC9tetra locus was mutated only in cancerous tissue, although it was excluded by transcription. The corresponding genes and proteins were significantly downregulated in malignant tissues, particularly in tumors with MSI. Furthermore, the promoter methylation results of the adenosine triphosphate binding cassette subfamily C member 9 (ABCC9) showed that there was a high level of methylation in breast tissues, but only one case showed a significant elevation compared with the control. In conclusion, MSI-Low or MSI-Stable is characteristic of most sporadic mammary tumors. Genes associated with tumorigenesis are more likely to develop MSI. ABCC9 protein and transcription abnormalities may be associated with ABCC9tetra instability.

## Introduction

Tumorigenesis is a complex multistep process associated with metabolic abnormalities and genomic instability (1). Studies have shown that tumor cells differ significantly from normal cells in terms of ion channel expression activity and membrane potential (2, 3). Through electrochemical synapse ionic coupling networks, tumor cells can induce or inhibit the occurrence and metastasis of tumors (4). The adenosine triphosphate (ATP)-binding cassette subfamily C, member 9 (ABCC9) can be matched with potassium channel proteins Kir6.1 (KCNJ8) or Kir6.2 (KCNJ11) to assemble ATP sensitive K^+^ channels (K_ATP_) in the heart, pancreaticislets, skeletal muscle and smooth muscle (5). The K_ATP_ channel is controlled by G proteins and allows potassium to flow into the cell. Previous studies have found that blocking the activity of K_ATP_ channels can significantly inhibit the proliferation of glioma and xenografted cells, inhibit the cell cycle at the G0/G1 phase, and induce apoptosis (6, 7). In contrast, the opening of K_ATP_ located on the mitochondrial membrane can attenuate cell apoptosis by maintaining the mitochondrial membrane potential (8).

As short tandem repeat DNA motifs (1–6 bp), microsatellites (MS) are ubiquitous in the eukaryotic genome, and the mutational rate of insertions/deletions in MS sequences is 10–100 times higher than that of traditional gene coding sequences. In 1993, cancer geneticists first discovered loss of heterozygosity (LOH) and microsatellite instability (MSI) in colorectal tumor tissues as a result of DNA mismatch-repair pathway obstruction, revealing a new pathway for oncogenesis (9). A previous study revealed that MSI is associated with clinical and pathological features in tumor tissues (10). Patients with the MSI-positive phenotype have a more robust T lymphocyte response than microsatellite-stable (MSS) cancer patients (11, 12). In addition, recent studies have shown that the diagnosis of MSI is tissue-specific, with varying frequency and prognostic values across multiple cancer types (13–15).

Mammary tumors as the common disease in female dogs. The MSI in canine mammary tumors (CMTs) has not been well-studied. Therefore, the aim of this trial was to investigated the relationship between MSI and tumor formation by screening MS loci in CMTs.

## Methods

### Material Collection and Histopathology Examinations

Twenty-two CMTs from different breeds of female dogs were provided by the Teaching Hospital of Nanjing Agricultural University. Procedures were approved by the Animal Ethics Committee of Nanjing Agricultural University (NJAU - 20171019, 10 October 2017). Experiment operates were performed under the Guidelines for Care and Use of Laboratory Animals of Jiangsu province (SYXK2017 - 0027). The mean age of the 22 canine patients was 9.77 ± 0.50 years, and the main breed was poodles (7/22, 31.8%). The adjacent normal and mammary gland tumors were excised; half of the samples were fixed in 10% formalin solution, and the remaining samples were stored at −80°C for further DNA and RNA extraction. The fixed samples were processed in a series of graded ethanol solutions and cleared with xylene. The samples were then embedded in paraffin, sectioned at 4 μm thickness, and stained with hematoxylin and eosin. Each stained tumor and its matched non-neoplastic tissue were examined using light microscopy.

### DNA Extraction and Microsatellite Locus Identification

DNA was isolated using the Animal Tissues/Cells Genomic DNA Extraction Kit (Solarbio Science & Technology Co., Beijing, China). Based on the instructions, 25 mg of tissue sample was used. The concentration and purity were estimated using a NanoDrop 2000 (Thermo Fisher Scientific, Waltham, MA, USA). The polymerase chain reaction (PCR) was performed using 500 ng of total DNA and TaKaRa Premix Taq™ according to the manufacturer's recommendations (Takara Co., Otsu, Japan). Genomic microsatellite loci were identified as described in our previous study (16), Table 1 shows the 58 pairs of primers used in this research. The cycle conditions were as follows: an initial incubation of 94°C for 5 min followed by 30 cycles of 30 s at 94°C, 30 s at their Tm (56–60°C), 30 s at 72°C, and finally extension at 72°C for 10 min. PCR amplified fragments were separated by 10% denatured polyacrylamide gel electrophoresis for 8 h at 100 V; mutations were observed by silver staining.

**Table 1:** Primer informations.

**Type**	**Primers**	**Genomic location**	**Amplicon size (bp)**
**Microsatellites**			
FH2305	F:TCATTGTCTCCCTTTCCCAG	CFA 30	208
	R:AAGCAGGACATTCATAGCAGTG		
CDK6B	F:TTGGGGCCAGATGTTGTTAG	CFA 14	285
	R:GAAGGAAAAGAGAAACAAGGCAA		
AHTK209	F:AGTGGTAGGTGTTCCAGCCG	CFA 20	91
	R:TCGACCTCTTGAGATAACAA		
C22.763	F:CAGCCCACTTCCTGGAAATA	CFA 22	206
	R:GACCAGTGTGCATTAAGCC		
AHT117	F:GCCTGCGTGGTACACACACA	CFA 1	84
	R:GTTTACCTGCCATCATCTCA		
REN287B11	F:CAGATTCCAGGTTGGGAAGA	CFA 5	348
	R:AGCTGTAGGATACGCCGAGA		
REN122J03	F:GTGCGAGTCATCAACAAAT	CFA 5	197
	R:ACTAAAGCCCATAAATCGTG		
FH3837	F:GGCCTCGTAGAATACATTTGG	CFA 5	325
	R:AGCAAGGAAGGCATCTGG		
CDK6A	F:TAACTTTTATTTATTTATGATA	CFA 6	163
	R:GGCGCCTGCCTTTGGCCCAG		
ABCC9ca	F:TCCAAGGTTTGTGTAAGGGT	CFA 27	240
	R:GGATTCAAGGTATATGCCCA		
HIVEP3	F:ACAGTCAAGGGTGCAAGAA	CFA 15	264
	R:ATGGCTCAGCGGTTTAGTGT		
TBC1D5	F:TGCCAGGCAATTACAAAAGA	CFA 23	291
	R:GCAGAAATCCTTGAAGCCAG		
ORF133	F:TACTTCTGTGTTCATCATCC	CFA 12	333
	R:GCTTTATTCAAGTATGCTTA		
REN49F22b	F:GGGGCTCTGTTATTAGGTG	CFA 22	154
	R:TCATAAGGCAAAGAAAACC		
15F11	F:TCTGGCTAGAGGTTTATCCA	CFA 6	234
	R:ACACAGGCCTAACTCAAGAA		
REN41F10	F:TACCCCAATGTTTACTGC	CFA 2	221
	R:TATTTGTCTATTTTGCTCTGA		
SLCA4	F:TATGCCTTGAGACTTCATCC	CFA 13	168
	R:CCAGAAAGAATCTAATCCCAC		
DKFZ	F:CTGGATCCTTTTCCTGTGGA	CFA 34	132
	R:AGGACACCTGTTGTTCTTGG		
FLJ32685	F:CTGCCTCAGCTGGGAAAATA	CFA 23	436
	R:CACTACAGCTGGGATCAGCA		
LRFN2A	F:TGGTTCAGTTCGTTGAGTGC	CFA 12	316
	R:ATGTCTGTGGTGACGCAAAA		
WNT2B	F:TGATACTGCCAGTCAGCAGG	CFA 17	277
	R:GAGGGAGGAGAACCTTGGTC		
LPP	F:TCAGTGAGGCAGATTTGGTG	CFA 34	415
	R:CAAACGCCTTGCTTCTTGTC		
MLH1	F:GGTTTAGTGCCGCCTTCAC	CFA 1	297
	R:GAGAAATGCTATGTGGCAAA		
REN47D17	F:GGCACTTGAGCTCTAATCCTA	CFA 1	346
	R:TGCTAATGAATCCACAGAATG		
REN47J11b	F:TCTCCTCGCGTGTTTCTG	CFA 18	170
	R:GGGGACACTCAGAAGGACG		
C26.733	F:CCCTCTACTTATGTCTCGGCC	CFA 26	255
	R:GAGAGGAGAAACAACCAACACC		
CXX.279	F:TGCTCAATGAAATAAGCCAGG	CFA 22	128
	R:GGCGACCTTCATTCTCTGAC		
C08.410	F:GAGGAAAACCAAGTGATTTTGG	CFA 8	114
	R:ACCTGCAAGTGACCCTCTCT		
FH2516	F:AATGGATGGAACTTAGGGCA	CFA 36	190
	R:CTGCATCTGGTAACCATCGA		
TRERFI	F:TTTGACCCCCCAAATGATAAA	CFA 12	164
	R:CAACCGCTAAGCCACTCAG		
MAML1	F:GTGATCCTGGAGTCCCGGAA	CFA 11	212
	R:CACACAATGTCACGGAGGAGG		
TPK1	F:AAACATACTTTTCTACATGGTT	CFA 16	167
	R:TTGTAATTGTGACAGATCATAG		
RYR3	F:CATGCAGATGCCCCTAATCT	CFA 30	165
	R:GGTGACAGGTGATTCTTGGA		
CXX873	F:CTGGCAGATTACAGGTAGC	CFA 11	145
	R:GTTCTCCAAAGCACTCAT		
C01.424	F:AGCTTAGCTTACTGCCCTGG	CFA 1	176
	R:TCCTTTGGTTTTTAGCAGGG		
HLA	F:ATCAACAATGCATGCCACAT	CFA 7	407
	R:GAGGAGGTGGGGAGATTGGC		
CPH14	F:GAAAGACAATCCCTGAAATGC	CFA 5	193
	R:ACCCCATTTATGAGAATCATGT		
ABCC9tetra	F:GCATTAAGGAGGGCACTTGA	CFA 27	219
	R:TAAGACCCAGCCTTGA		
FH2060	F:GTTTTGAGGAAGCCTTGCTG	CFA 14	222
	R:GAAGGAAGGGGCCAGTATTC		
SCN10A	F:TCCAAGCATCCTCTTATCCA	CFA 23	196
	R:CCACGTTGGTCTCCCTACTTA		
ANGPT1	F:GTTTTCCTGCTGTCCCAGTG	CFA 13	390
	R:TTCCCTTTTGTGAATCCTGC		
SCN11A	F:GCAGTTTGGGGACTGCTAAA	CFA 23	260
	R:AGAATGGAATCTTGCCCAGA		
IGHE	F:CAAGACTGGCTCTGCTCTG	CFA 8	141
	R:CCACTGAAAACAAGCCCATC		
CDH4	F:AAGTCAACAAGCTCCATCCC	CFA 24	136
	R:AGGATTTTCCCCTAAGAGCTG		
PPP1R9A	F:TAAAGATCCAAGTGGCGAGG	CFA 14	189
	R:AACCACTCCCTTCACCACAG		
9A5	F:GTCTGCTTTCAACTCAGGTC	CFA 4	266
	R:CTCTAAACTGGACTTCGTGG		
FH2401	F:CTGATTCTGCCCATTGGG	CFA 12	224
	R:ATGTAAGCTCTACTGGGGTACTGG		
FH2377	F:TCCCTTGGGGAAGTAGAGTG	CFA 34	312
	R:TAGCTAATGTGGTTAACGGTTACC		
REN198P23	F:TTGTACATTATCTGTTCTACCTCGG	CFA 9	132
	R:TCTTCAGCAGGCCTTTTCTC		
AHT137	F:TACAGAGCTCTTAACTGGGTCC	CFA 11	137
	R:CCTTGCAAAGTGTCATTGCT		
FH3113	F:CTGAATTATGGGAAAACATGG	CFA 5	207
	R:CAGGGAAGGAAGAAAACAGC		
FH2594	F:TTTAAGGAGCTGCTCATGCA	CFA 5	311
	R:CTGAAATTCCTGGCCCAGTA		
FH2561	F:TGCTCAAGGTTGAATAAATATGC	CFA 6	364
	R:TTTATGGCCTGTGGGCTC		
FH2175	F:TTCATTGATTTCTCCATTGGC	CFA 16	253
	R:AGGACTCTAAAAACTTGCCTCC		
FH2495	F:ATTTCATATGTGAGGCTGAGATTG	CFA 24	132
	R:CAGTGGGAGAAAGATGCCAT		
BTN1A1	F:CTGCCATGTAGGGTGTTT	CFA 12	240
	R:ACCCTTTGACAAGAGCTC		
CPH5	F:TCCATAACAAGACCCCAAAC	CFA 17	114
	R:GGAGGTAGGGGTCAAAAGTT		
C13.900	F:TTGGACTTCTAATTTTTCATT	CFA 13	128
	R:CAACTGACTAAATCTCCTAATG		
**Genes**			
*GAPDH*	F:ACCACAGTCCATGCCATCAC	U94889	268
	R:CCTGCTTCACCACCTTCTTGA		
*A5B*	F:GCACGGAAAATACAGCGTTT	NM_001686	187
	R:TTGCCACAGCTTCTTCAATG		
*HPRT*	F:TGCTCGAGATGTGATGAAGG	NM_000194	192
	R:TCCCCTGTTGACTGGTCATT		
*β-actin*	F:GATATCGCCGCGCTCGTCGTC	U39357	384
	R:GGCTGGGGTGTTGAAGGTCTC		
*RPS5*	F:GGATGACCGAGTGGGAGA	XM_022427163	122
	R:TGCAATGTAGTCCTGCAAAGA		
*RPL8*	F:AGGTCATTTCTTCCGCCAA	XM_853403	164
	R:AGGATGCTCCACAGGATTCA		
*RPL32*	F:ATGCCCAACATTGGTTATGG	XM_540107	181
	R:CTCTTTCCACGATGGCTTTG		
*ABCC9*	F:TGTGCATCATCTGTTTTTGTGCT	NC_006609	183
	R:TTAGGGCCTGCTATGGGCTA		
**BSP analysis**			
ABCC9-1	F:AGAGTGGAGGAGGGAGAAGTAGGTTTTATG		265
	R:CAAACAATCCCCRAACACACACCTAAATATC		
ABCC9-2	F:GTGATAAATAGTTTYGGGGGGTAGTTGG		228
	R:ACCTAAAAAAACTAAAACCRACCCCCCC		

MSI was defined as addition or deletion of fragments to one or both tumor DNA alleles compared with normal tissues; LOH was defined as a reduction in the DNA signal intensity of tumor allele at least 50% (17). Positive cases were repeated three times to confirm the results. For MSI identification, mutation products were purified and cloned into the pMD19-T vector (Takara Co., Otsu, Japan) and sequencing. Sequence alignments were conducted using DNAMAN software v9.0.1.

### RNA Extraction and mRNA Analysis

Total RNA was isolated using the Total RNA Extraction Kit (Solarbio Science & Technology Co., Beijing, China). Based on the instructions, 100 mg of tissue sample was used. The size of the RNA samples was estimated using a NanoDrop 2000 (Thermo Fisher Scientific, Waltham, MA, United States). Reverse transcription was performed using 800 ng of total RNA treated with DNase I and PrimeScript RT Master Mix Perfect Real Time according to the manufacturer's instructions (Takara Co., Otsu, Japan).

The mRNA levels of MSI-mutated adjacent genes were detected by quantitative real-time PCR (QRT-PCR). Target sequences were amplified using Green Fast qPCR Mix (Takara Co., Otsu, Japan) and analyzed with an ABI 7300 instrument (Applied Biosystems, Foster City, CA, USA). The primer information is presented in Table 1. The cycle conditions were as follows: 95°C for 15 s followed by 95°C for 5 s and 60°C for 31 s for 40 cycles. The specific of each gene primer was confirmed by melting curve performance and gel electrophoresis. Results were presented as CT mean values of three technique replicates.

Reference genes were evaluated using geNorm v3.5 and NormFinder v0.953. Finally, the geometric means of *A5B, RPL8, GAPDH, RPL32, RPS5*, β*-actin*, and *HRPT* were used for normalization.

### DNA Methylation Analysis

DNA methylation was measured using next-generation sequencing based bisulfite sequencing PCR (18). First, DNA modification with sodium bisulfite of 6 canine breast cancers and matched samples was performed using an EZ DNA Methylation Kit (Zymo Research, Irvine, CA, USA) according to the manufacturer's protocol. The sequence included 2,000 bp upstream of the *ABCC9* transcription start site and 1,000 bp downstream (a total of 3 kb). Elution products were then used as templates for PCR amplification with 35 cycles using the KAPA 2G Robust HotStart PCR Kit (Kapa Biosystems, Wilmington, MA, USA). The primers for BSP were designed using online MethPrimer software (Table 1). The bisulfite sequencing PCR products of each sample were pooled equally, 5'-phosphorylated, 3'-dA-tailed and ligated to a barcoded adapter using T4 DNA ligase (Thermo Fisher Scientific, Foster city, CA, USA). The barcoded libraries were then prepared and sequenced on an Illumina platform. Using the clean sequencing reads directly aligned to the target sequences, Bsmap v2.73 software was used with the default parameters. Methylation level was defined as the fraction of “C” read counts in the total read counts of both “C” and “T” for each covered C site. According to the method of Lister (19), each methylation context calculates the probability mass function, and only those CGs covered by at least 200 reads in one sample were considered for testing.

### Immunohistochemical Analysis

Tissue sections were taken from 22 CMTs along with adjacent controls after fixation in 4% paraformaldehyde, dehydration, and embedding in paraffin. The expression of ABCC9 (1:200, Affinity Biosciences Cat# DF9255) in the breast was examined using an SP immunohistochemistry kit (Sangon Biotech Co., Shanghai, China) according to the manufacturer's instructions. A semiquantitative determination was conducted with Image J software to detect protein expression. The immunohistochemical staining intensity was expressed in average optical density (AOD) units, AOD = integrated optical density (IOD)/Area; five fields were randomly selected in a blinded manner, counted for the signal density of tissue areas, and then statistically analyzed.

### Data Analysis and Statistics

The statistical analyses were conducted with GraphPad Prism software version 8.0 and SPSS version 21.0. A genome map of microsatellite loci was constructed using the MapChart program. The comparison of results between MSI/LOH and tumor type and methylation data were performed using Fisher's exact test. The Mann–Whitney *U*-test was used to analyze vs. benign and breast cancer groups. The relative mRNA expression levels of *ABCC9* in tumors and matched normal tissues were calculated using the 2^−ΔΔCt^ method. The *t*-test was performed to compare the relative mRNA level and protein expression between the two groups. The results are presented as the mean ± SD. The statistical significance was set at *p* < 0.05 for all analyses.

## Results

### The Pathological Identification of CMTs

**Figure 1:**
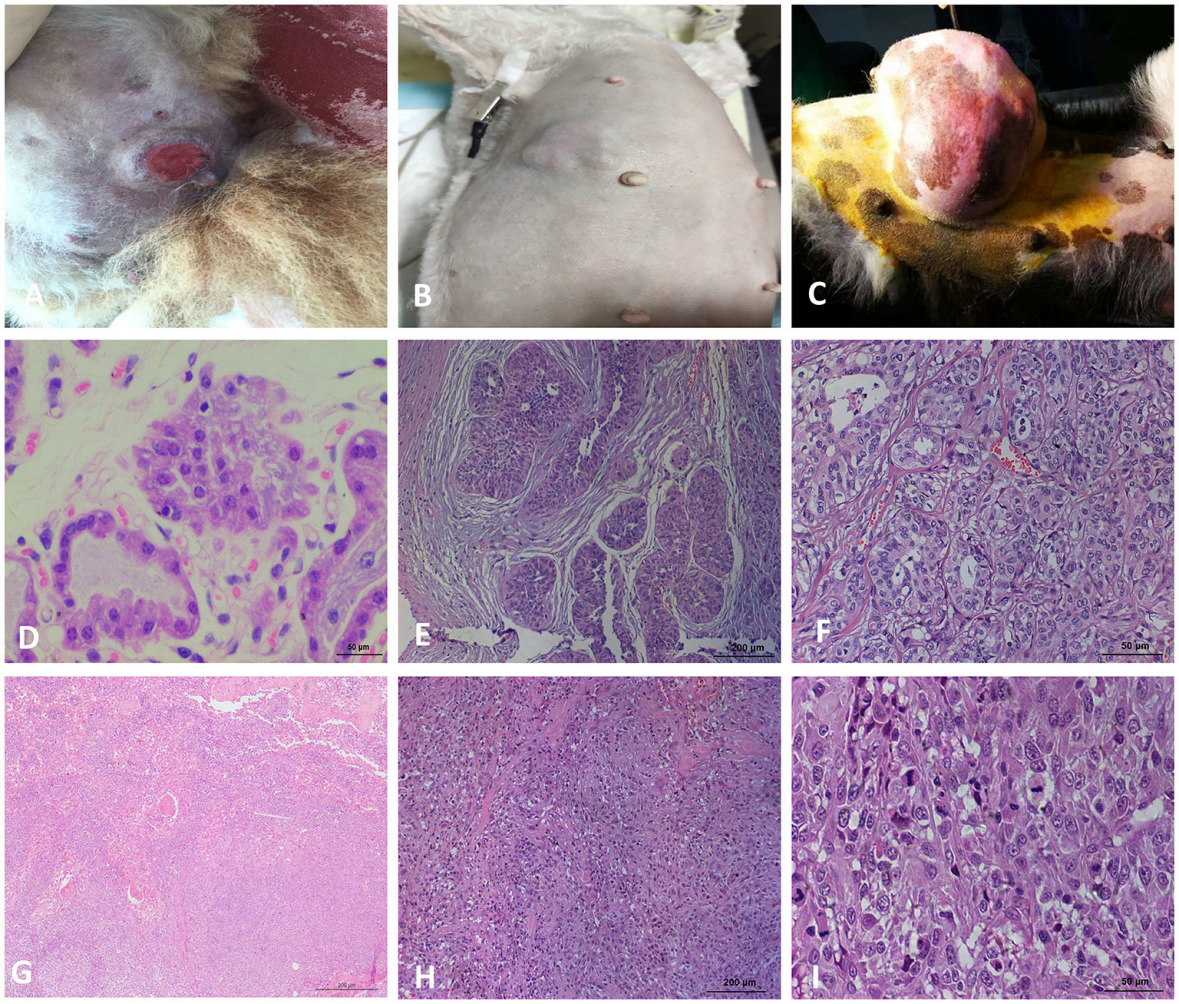
Macroscopic observation and HE staining of CMTs. **(A)** Macroscopic observation of CMT, the skin surface of the tumor ruptured. **(B)** Macroscopic observation of CMT, a cauliflow-like mass in mammary gland with obviously boundary and hard texture. **(C)** Macroscopic observation of CMT, the tumor located on the mammary tissue with a great size. **(D)** HE staining of the breast lobules in a normal dog (400×). **(E)** HE staining of mammary gland adenoma (200×), the capsule is intact and tumor cells grow in the enlarged lumen. **(F)** HE staining of mammary gland adenoma (400×), Adenoma arising in the glandular tissue, myoepithelial cells are inconspicuous, the islands of neoplastic cells are separated by a fine fibrovascular connective. **(G)** HE staining of solid carcinoma (200×). **(H)** HE staining for ductal carcinoma (200×), tumor cells invaded the connective tissue, glandular ducts were disappeared. **(I)** HE staining for ductal carcinoma (400×), tumor cells are pleomorphic and mitotic.

The 22 CMTs were classified as either benign (8/22, 36.4%) or malignant (14/22, 63.6%) (Figure 1). Based on the predominant cell type, the benign tumors were subclassified as fibroadenoma (4/8, 50%), complex adenoma (1/8, 12.5%), adenoma (1/8, 12.5%), or intraductal papilloma (2/8, 25%). The malignant tumors were subclassified into invasive ductal carcinoma (7/14, 50%), situ carcinoma (1/14, 7.1%), ductal carcinoma (1/14, 7.1%), complex carcinoma (2/14, 14.3%), intraductal papillary carcinoma (2/14, 14.3%), or solid carcinoma (1/14, 7.1%).

### Malignant Tumors Have More MS Mutation Loci Than Benign Tumors

**Figure 2:**
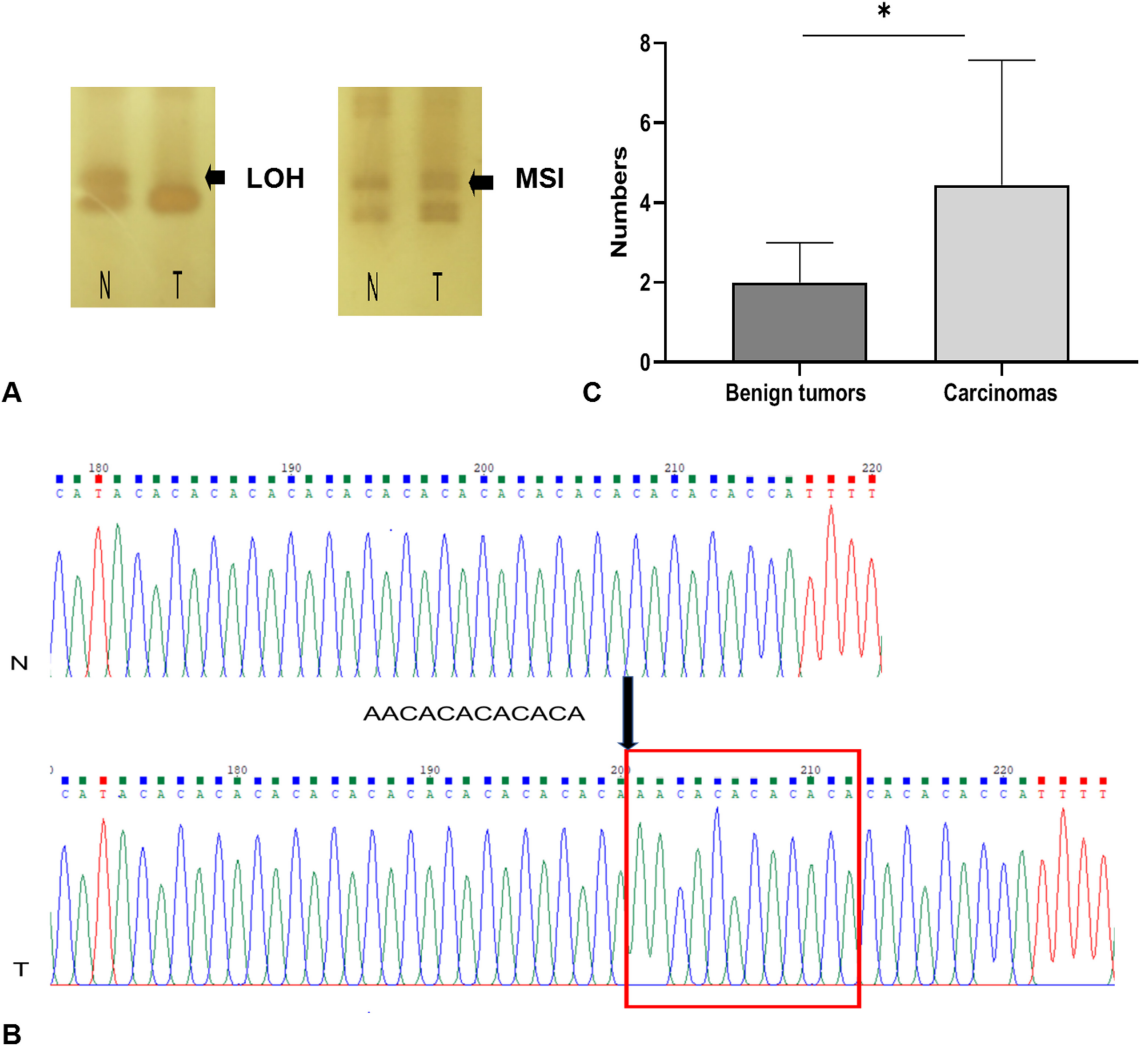
MSI and LOH occurring in CMTs. **(A)** MSI and LOH detection in denatured polyacrylamide gels. **(B)** The sequencing result of MSI locus, there was a repetitive fragment insertion in tumors. **(C)** Carcinomas have more mutation loci than benign tumors. *indicates a significant difference between the two groups, *P* < 0.05.

Using the panel of 58 MS markers, a LOH/MSI analysis between tumor tissues and their matched non-neoplastic tissues was carry out, the variation in the electropherogram of MS makers was described in Figure 2A. Differential bands were extracted and sequenced (Figure 2B). The sequencing result verified that the mutation form of MS in CMTs mainly included the insertion or deletion of nucleic acid fragments in repeated sequences. In addition, point mutations were also discovered in flank conserved sequences of MSI loci. Based on the National Cancer Institute guidelines (20), 14 tumors (14/22, 63.6%) were defined as MSI-L (MSI-Low), and 8 tumors were defined as MSS (MSI-Stable) (Table 2). Of the MSI-L tumors, 5 were diagnosed as benign tumors, and 9 were diagnosed as breast cancers. In addition, we found that the phenomenon of LOH was present in 6 MSI-L tumors (6/14, 42.9%), of which 2 tumors were benign and 4 tumors were malignant. There was no evidence of a difference in mutation rates between MSI and LOH in benign or malignant tumors (Fisher's test, *P* > 0.05). However, the histological type was significantly correlated with the number of MSI loci. Malignant tumors had more MS mutation loci than benign tumors (*P* < 0.05) (Figure 2C). Case 13 had the highest frequency of MSI (10/58, 17.2%) in this study, which was defined by pathological grading as grade III.

**Table 2:** Information of canine mammary tumors.

**Case no**.	**Age**	**Breed**	**Tumor Histo-type**	**MSI/LOH mutation**	**Tumor type**
1	8	Pomeranian	Intraductal papilloma		MSS
2	9	Golden retriever	Invasive ductal carcinoma	MSI	MSI-L
3	13	Mongrel dog	Invasive ductal carcinoma	MSI	MSI-L
4	8	Poodle	Situ carcinoma	MSI/LOH	MSI-L
5	12	Pomeranian	Complex adenoma		MSS
6	11	Poodle	Adenoma	MSI/LOH	MSI-L
7	11	Mongrel dog	Ductal carcinoma	MSI	MSI-L
8	5	Poodle	Fibroadenoma		MSS
9	9	Schnauzer	Complex carcinoma		MSS
10	8	Bichon Frise	Intraductal papillary carcinoma	MSI	MSI-L
11	11	Rottweiler	Complex carcinoma	MSI/LOH	MSI-L
12	9	Samoyed	Fibroadenoma	MSI	MSI-L
13	12	Border Collie	Solid carcinoma	MSI/LOH	MSI-L
14	10	Poodle	Invasive ductal carcinoma	MSI/LOH	MSI-L
15	7	Poodle	Fibroadenoma	MSI	MSI-L
16	9	Poodle	Intraductal papillary carcinoma		MSS
17	13	Mongrel dog	Invasive ductal carcinoma	MSI	MSI-L
18	10	Samoyed	Invasive ductal carcinoma		MSS
19	5	Poodle	Invasive ductal carcinoma		MSS
20	13	Mongrel dog	Intraductal papilloma	MSI/LOH	MSI-L
21	10	Yorkshire	Invasive ductal carcinoma		MSS
22	12	Mongrel dog	Fibroadenoma	MSI	MSI-L

### Tetranucleotide Microsatellites Are Prone to Instability in CMTs

**Figure 3:**
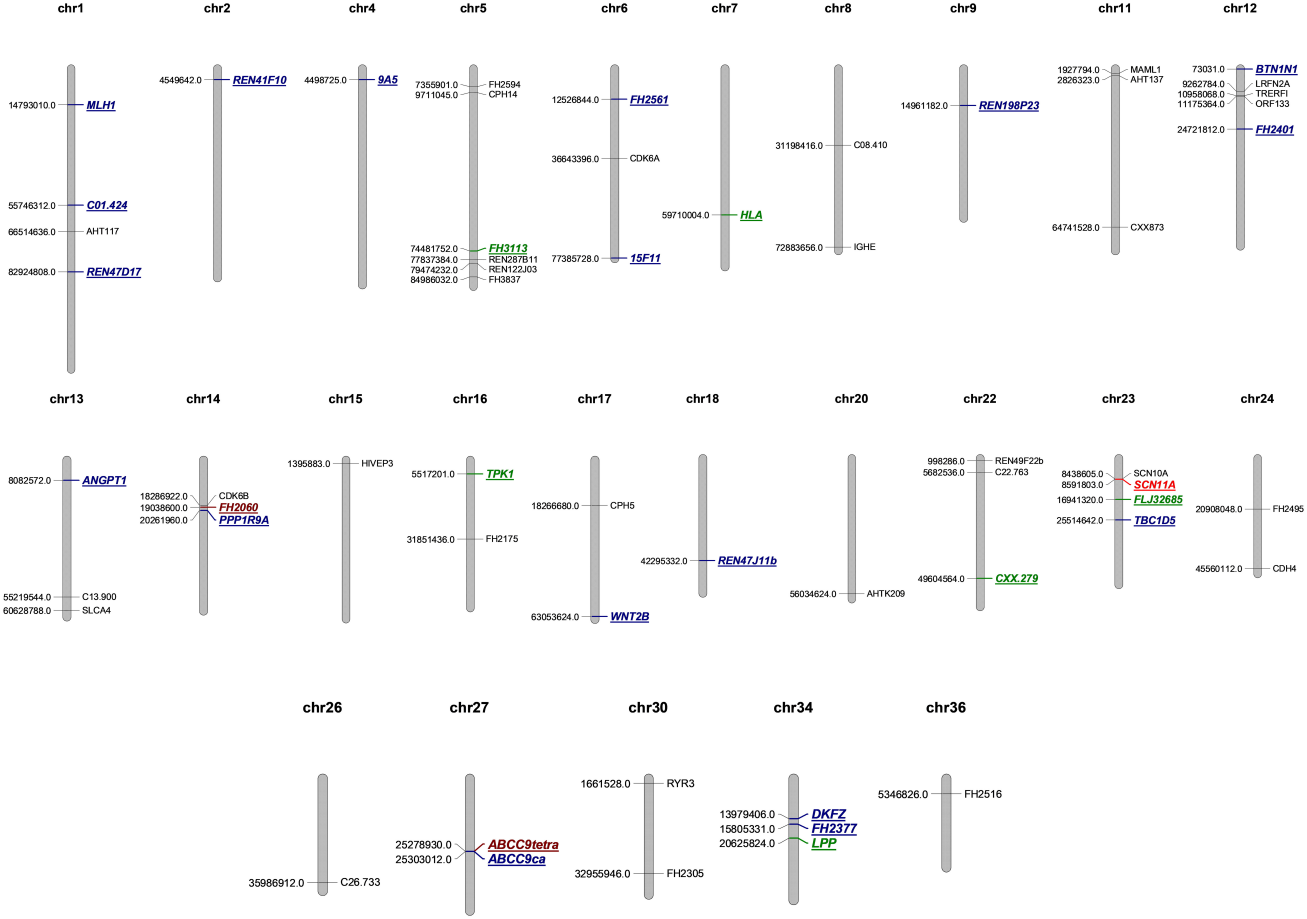
Genome map of microsatellite loci in this study. MSS are depicted in black, the single mutation of microsatellite loci is labeled blue, twice mutation is green, four times mutation is brown, and six is marked in red.

A total of 44 aberrations of MSI were found at 27 MS loci (27/58, 46.5%), which were distributed across 17 chromosomes (Figure 3). The classification of mutated MS markers in this study was shown in Table 3. In addition to dinucleotide [CA]n, tetranucleotide [CTTT]n and more complex types of microsatellite loci also has a high mutation frequency in this research. Among them, most of MS loci were only mutated once (1/14, 7.1%). The interrupted marker SCN11A (6/14, 42.9%) and tetranucleotide markers FH2060 (4/14, 28.6%) and ABCC9tetra (4/14, 28.6%) were loci with high mutation rate from the result. Moreover, the phenomenon of LOH was also observed on FH2060 (4/6, 66.75%), SCN11A (2/6, 33.3%), ABCC9tetra (1/6, 16.7%) and PPP1RA (1/6, 16.7%). Table 4 shows the mutation results for ABCC9tetra, FH2060 and SCN11A markers in CMTs. There were five tumor cases had at least two loci mutated as MSI or LOH for ABCC9tetra, FH2060 and SCN11A. Because of the locus of ABCC9tetra was only mutated in malignant group, the relationship between ABCC9tetra and breast cancer were studied.

**Table 3:** Classification of mutated microsatellite DNA markers.

**Motif**	**Marker**	**Sequence**	**Number**
Pure Di	TBC1D5	[CT]_21_	1
	TPK1	[CA]_20_	2
	C01.424	[CA]_13_	1
	MLH1	[CT]_21_	1
	ABCC9ca	[CA]_19_	1
	REN47D17	[CA]_16_	1
	15F11	[CA]_10_	1
	CXX.279	[CA]_17_	2
	REN47J11b	[CA]_11_	1
	WNT2B	[CA]_21_	1
	REN198P23	[CA]_15_	1
	DKFZ	[CT]_20_	1
	Ren41F10	[CA]_20_	1
Pure Tetra	FH2060	[AATG]_5_	4
	FH2377	[CTTT]_4_	1
	PPP1R9A	[GTTT]_9_	1
	9A5	[CTTT]_18_	1
	FH2401	[CTTT]_11_	1
	FH2561	[CTTT]_21_	1
	HLA	[CTTT]_13_	2
	ABCC9tetra	[CTTT]_8_	4
Pure Penta	BTN1A1	[CTTTT]_3_	1
Compound Tetra	LPP	[TTCC]_5_[CTTT]_15_	2
	FLJ32685	[CTTT]_14_[CTTTT]_14_	2
Interrupted	SCN11A	[CAAT]_3_[CTAT]_4_ CATC[TATC]_5_	6
	FH3113	[TG]_7_ A[GT]_3_[ACGC]_2_	2
	ANGPT1	[CCTT]_12_T[CTTT]_11_	1

**Table 4:** Frequency of ABCC9tetra, FH2060, and SCN11A in MS mutation tumors.

**Type**	**Cases**	**ABCC9tetra**	**FH2060**	**SCN11A**
Benign	6	I	LOH	MSI/LOH
	12	I	I	I
	15	I	I	I
	20	I	LOH	MSI
	22	I	I	MSI
	Mutation frequency (%)	0	40	60
Cancer	2	I	I	I
	3	I	MSI	MSI
	4	MSI	MSI/LOH	I
	7	MSI	I	I
	10	I	I	MSI
	11	MSI	MSI	MSI/LOH
	13	I	MSI/LOH	I
	14	MSI/LOH	I	I
	17	I	I	I
	Mutation frequency (%)	44.44	44.44	33.33

*I indicated that LOH or MSI does not occur at this site, and LOH/MSI indicates that both LOH and MSI occur at this site*.

### ABCC9 Is Downregulated in Canine Breast Cancer

**Figure 4:**
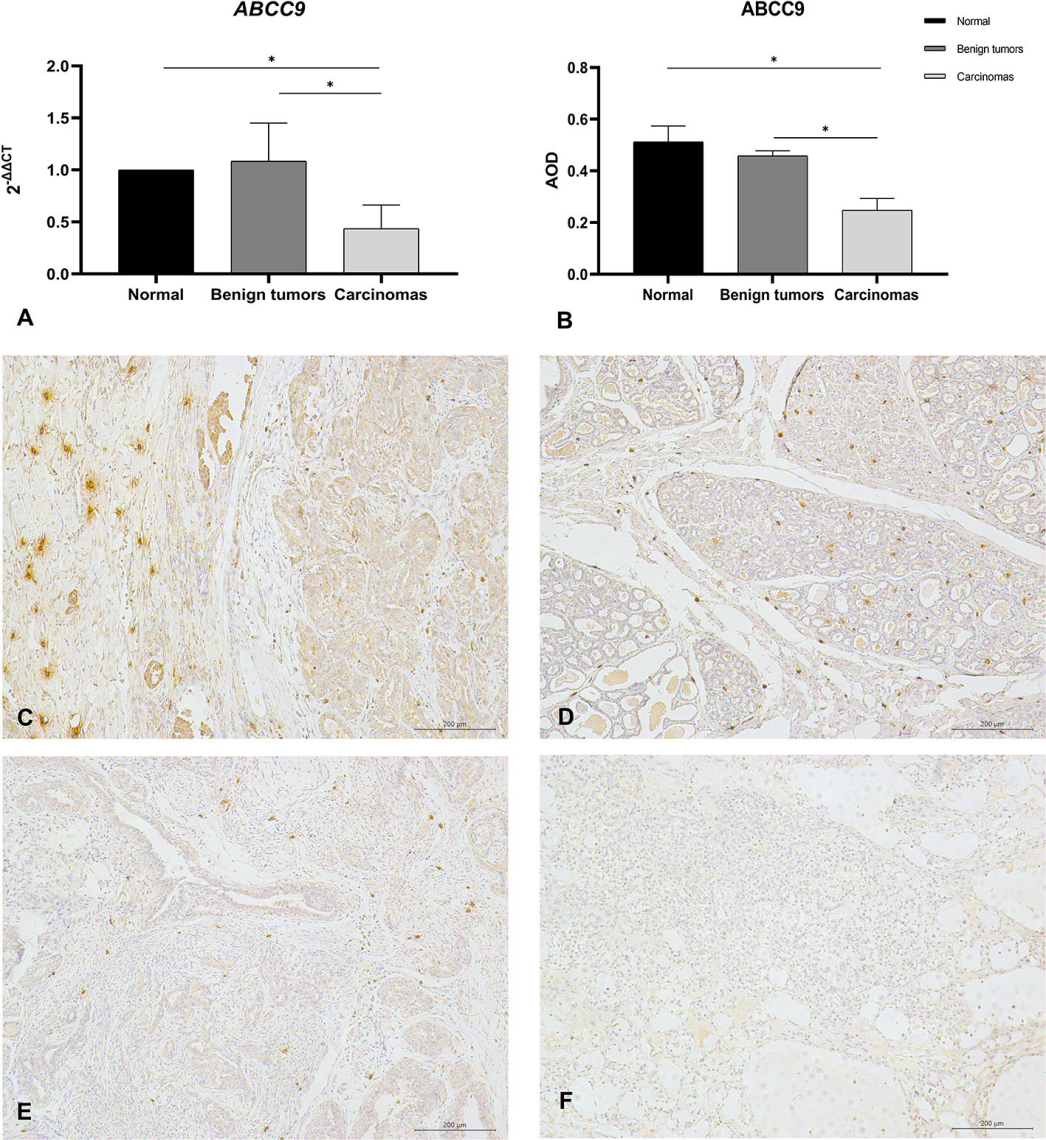
The mRNA level and protein expression of ABCC9. **(A)** The mRNA level of *ABCC9*, the mRNA level was expression by 2^−ΔΔCt^. **(B)** Average optical density values of ABCC9 protein in CMTs. AOD = IOD/Area, all of data are shown as means ± SD, ^*^indicates that there is significant difference between two groups (*P* < 0.05). **(C)** IHC staining of ABCC9 in paracancer tissue (200×). **(D)** IHC staining of ABCC9 in benign tumor (200×). **(E)** IHC staining of ABCC9 in complex breast cancer (200×). **(F)** No strong positive staining of ABCC9 in higher malignancy cells (200×). Strongly positive cells can be observed in para-cancer tissues and benign tumors, but was significant decreased in malignant tumors.

NCBI revealed that ABCC9tetra was located in the intron region of *ABCC9*, and the mutation in this locus did not cause a frameshift mutation in open reading frame. But the result of QRT–PCR showed that the mRNA level of *ABCC9* was significantly downregulated in the malignant group (*P* < 0.05) (Figure 4A). And the result of immunohistochemistry was similar to it. The AOD value showed that the expression of ABCC9 protein in malignant tumors was significantly lower than that in para-cancer tissues and benign tumors (*P* < 0.05) (Figure 4B). Strongly positive cells can be observed in normal and para-cancer tissues (Figure 4C) and even in benign tumors (Figure 4D). However, the number of ABCC9 positive cells was significant decreased in malignant tumors (Figure 4E). Moreover, the expression of ABCC9 protein may be related to the cellular composition and pathological grading. In the tumor sample of grade III, ABCC9 strongly positive cells almost disappeared, and were only weakly or micro-expressed in cells (Figure 4F). In addition, ABCC9 protein expression and mRNA levels were significantly reduced in tumor samples with ABCC9tetra locus instability (*P* < 0.05).

**Figure 5:**
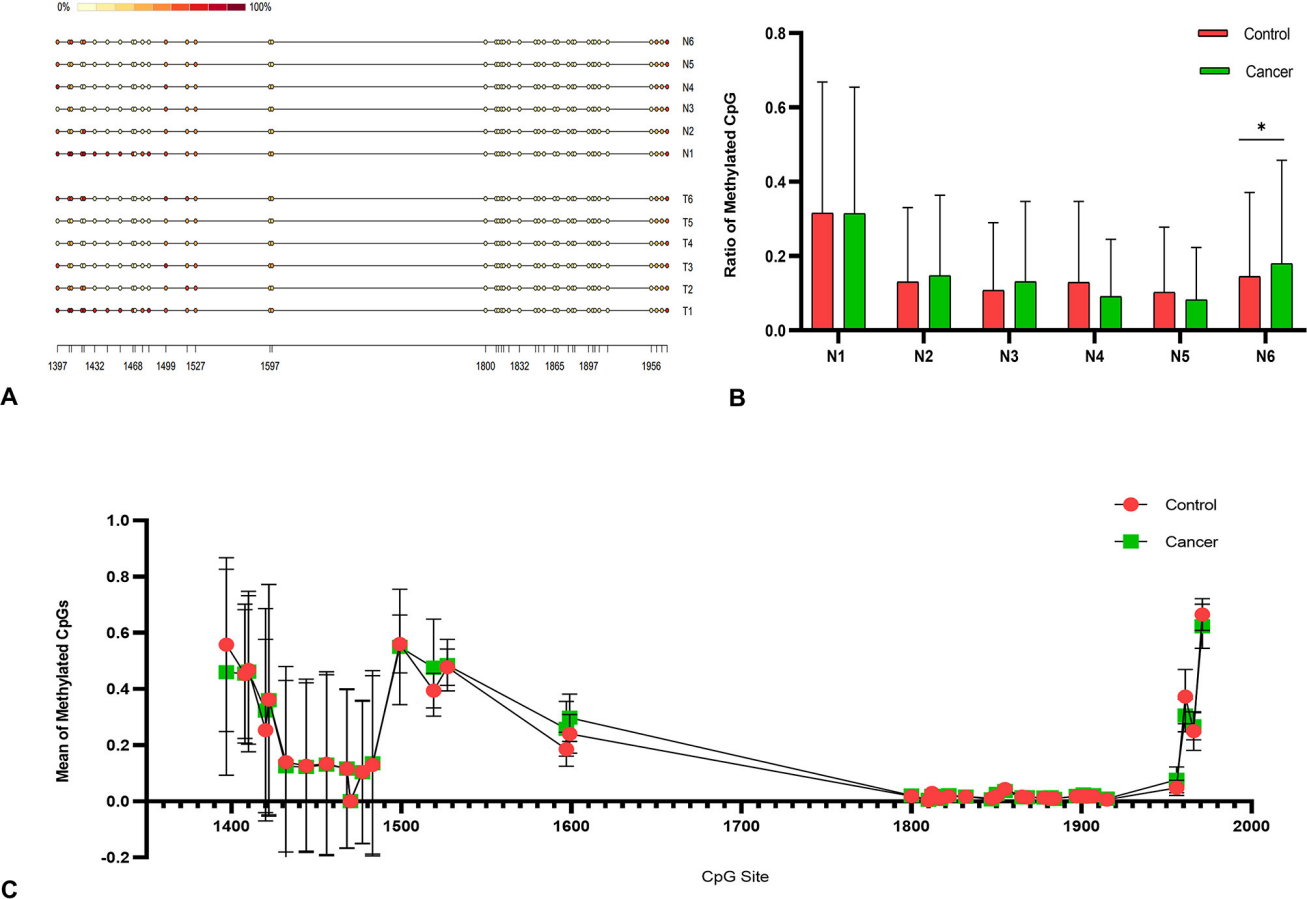
Methylation analysis of ABCC9 promoter. **(A)** Methylation analysis of ABCC9 CpG island. **(B)** Methylation CpG levels of six samples. **(C)** Methylation levels in average CpG sites. *indicates a significant difference between the two groups, *P* < 0.05.

Total of 6 tumor sample with *ABCC9* mRNA levels significantly reduced were tested by methylation analysis. MathPrimer software detected a 703 bp CpG island in *ABCC9* 5′UTR (GC = 65.4%, and Obs/Exp ratio = 0.92). The methylation results of *ABCC9* promoter CpG island revealed that high levels of methylation occurred at multiple sites in cancer tissues, but no new methylation sites were formed (Figure 5A). There was no significant difference in methylation level of each site (Figure 5C). And only one cancer sample showed significantly higher promoter methylation level than the control tissue (*P* < 0.05), with both MSI and LOH (Figure 5B).

## Discussion

Genomic instability is a hallmark of tumors, and tumor tissue has a higher mutation rate than non-tumor tissue. Study showed that the sensitivity of MSI detection is not limited by tumor heterogeneity or normal tissue contamination when large resection tissues are used (21). The most endorsed explanation of MS mutagenesis is the slip strand mispairing model, and repeated numbers of motifs are highly polymorphic among individuals. A previous study of an MS mutation model showed that deletion is produced by the misalignment loop in the template chain, and insertion is subsequently produced in the nascent chain (22, 23). According to the sequence alignment analysis, we found that MS mutations mainly included the insertion or deletion of repeat sequences and point mutations of flanking conserved sequences. In addition, in the same MS locus, the forms of the mutations were differed among the samples. This phenomenon may be due to the mutation of MS occurring at different stages of tumor cell replication, whereas the point mutation may be caused by the suppression of mismatch repair genes. The length and unit type of MS and DNA shape are the main factors influencing DNA fragility and have the greatest influence on the mutation rate (24). In addition to dinucleotides, tetranucleotides and interrupted MS also showed frequent mutations in our research, which confirmed the susceptibility of the DNA structure to mutation.

The guidelines of the National Cancer Institute suggest that MS that display instability at ≥ 2 loci or instability at ≥ 30–40% of loci (more than five loci) be defined as MSI-High (MSI-H). If all tumor MS loci are comparable to their normal specimen, the tumor is classified as MSS. The range between MSS and MSI-H is defined as MSI-L (20). To date, tumors with an MSI-H frequency of 0% and tumors with MSI mutations all exhibited the MSI-L type, which is consistent with studies by Eldama'ria and Ando (17, 25). Work by Dustin showed that 800 loci are required to achieve diagnostic sensitivity and specificity for HBC, and diagnosis using predefined microsatellite locus panels is challenging (26). Overall, 31 MS loci were stable, and 27 MS loci exhibited MSI. Different cancer types exhibited distinct patterns of MS mutations. It appears that for breast tumors, the instability event may have a more neutral fitness effect, resulting in fewer recurrent mutation loci.

Although there was no significant difference in the frequency of MSI or LOH between benign and malignant tumors, malignant tumors had more MSI mutation loci than benign tumors. Of the 23 that we previously reported (4 benign and 19 malignant tumors), ABCC9tetra, FLJ32685, SCN11A and 9A5 loci showed a higher incidence of instability events in most canine breast cancers (16). In the present work, ABCC9tetra (4/22, 18.2%) and SCN11A (6/22, 27.3%) loci also exhibited higher mutation rates in CMTs. Our newly discovered high-frequency MSI locus, FH2060 (4/22, 18.2%), also had the highest LOH frequency (4/22, 18.2%). This phenomenon is potentially caused by selective pressures in tumor evolution (14). Biological pressures are involved in the selection of MS mutations, and some specific MS may be subject to positive or negative selection through changes in gene expression or function that result in more malignant transformation such as proliferation and metastasis (27, 28). Furthermore, a previous study showed that LOH can confer a growth advantage in tumor cells, and the tumor suppressor genes *BRCA1* and *BRCA2* loci are frequently altered due to allelic imbalance during carcinogenesis in the breast (29). Therefore, we suspected that the MSI locus was involved in the formation of breast tumors and began to explore the genes adjacent to the MSI locus.

Cancer genome sequencing has revealed that regional autosomal differential mutation rates at megabase resolution are related to changes in the timing of DNA replication or in gene expression and are less correlated with cancer type (30). The effect of DNA damage on highly expressed genes is limited to the MS within a specific gene in a specific tissue. In our results, ABCC9tetra, an MS locus, was mutated only in malignant tumors. The expression of adjacent gene *ABCC9* was significantly decreased in the malignant tumor group. It is worth noting that the ABCC9 protein is involved in bioelectric control. ABCC9 can couples with potassium channel proteins KCNJ8 or KCNJ11 to form the K_ATP_ channel. The K_ATP_ channels were located on cell membranes and mitochondrial membranes. Past studies have shown that the channels formed by different combinations of KCNJ8, KCNJ11, ABCC8, and ABCC9 vary based on tissue localization (31). Immunohistochemistry reflected that ABCC9 was overexpressed in both normal and paracancerous tissues and in benign tumors, indicating that it is involved in the assembly of the K_ATP_ channel in the breast.

The ionic concentrations of Na^+^, K^+^, Ca^2+^, and Cl^−^ are regulated by ion channels. In this study, ABCC9 on cell membranes and the mRNA level of *ABCC9* were significantly decreased in malignant tissues. Furthermore, a negative correlation was observed between ABCC9 expression and cancer grading, with positive cells basically disappearing in cancer samples of grade III. This relationship may be due to the inhibition of the K_ATP_ channel in cancer tissue. The cytoplasm of depolarized cells is more positively charged relative to the extracellular space and has a less negative *V*_mem_ (32). Inhibition of potassium influx can lead to continuous depolarization of cells, which can induce mitosis and promote the proliferation of cancer cells (33, 34). Furthermore, a study of cardiac ischemia-reperfusion injuries revealed that the opening of mitoKATP channels could inhibit the depolarization of the mitochondrial membrane and protect against apoptosis in its early stages (35).

In addition, many studies have shown that ABCC9 can be used as a biomarker for cancers. The enrichment analysis of gastric cancer found that ABCC9 was involved in ATPase activity, transmembrane transport, and ABC transporters (36). Another study on the methylation pattern of breast cancer revealed that ABCC9 is a potential grade III biomarker of breast cancer in white individuals. However, in our study, only one case of cancer showed a significant increase in promoter CpG islands, which could not explain the reduced gene expression.

In conclusion, CMT is a highly heterogeneous disease with multiple genetic and epigenetic alterations. Malignant tumors have more unstable loci than benign tumors, which may be related to altered gene expression. ABCC9 is significantly downregulated in breast cancer and ABCC9tetra is particularly prone to mutation. In the future, additional studies on the regulation of ABCC9 protein in cancer cells are needed.

## Data Availability Statement

The datasets presented in this study can be found in online repositories. The names of the repository/repositories and accession number(s) can be found in the article/Supplementary Material.

## Ethics Statement

The animal study was reviewed and approved by the Animal Ethics Committee of Nanjing Agricultural University (NJAU-20171019, 10 October 2017). Experiment operates were performed under the Guidelines for Care and Use of Laboratory Animals of Jiangsu province (SYXK2017-0027). Written informed consent was obtained from the owners for the participation of their animals in this study.

## Author Contributions

PH and DWY: conceptualization. PH: methodology, software, formal analysis, resources, data curation, and writing-original draft preparation. SQW and XJH: validation. KYS: investigation. DWY: writing-review, editing and visualization. DJY: supervision, project administration, and funding acquisition. All authors contributed to the article and approved the submitted version.

## Funding

Natural Science Foundation of Jiangsu Province (BK20130686), National Natural Science Foundation of China (30871847), and Priority Academic Program Development of Jiangsu Higher Education Institutions (PAPD).

## Conflict of Interest

The authors declare that the research was conducted in the absence of any commercial or financial relationships that could be construed as a potential conflict of interest.

## Publisher's Note

All claims expressed in this article are solely those of the authors and do not necessarily represent those of their affiliated organizations, or those of the publisher, the editors and the reviewers. Any product that may be evaluated in this article, or claim that may be made by its manufacturer, is not guaranteed or endorsed by the publisher.
