# Supplementary material for: ABCC9 Is Downregulated and Prone to Microsatellite Instability on ABCC9tetra in Canine Breast Cancer
Source: Front Vet Sci. 2022 Jan 7;8:819293. doi: 10.3389/fvets.2021.819293 (PMC8777218; doi:10.3389/fvets.2021.819293)
Supplement: Supplementary file 1 [file Data_Sheet_1.doc]

<https://www.jianguoyun.com/p/DdUPaCAQzJWEChjJu5sE>

<https://www.jianguoyun.com/p/DSGbcToQzJWEChjMu5sE>

<https://www.jianguoyun.com/p/DaasvZ4QzJWEChjRu5sE>

<https://www.jianguoyun.com/p/DVdZT64QzJWEChjSu5sE>

<https://www.jianguoyun.com/p/DWbq5qgQzJWEChjTu5sE>

<https://www.jianguoyun.com/p/DctcOXAQzJWEChjUu5sE>

<https://www.jianguoyun.com/p/DZYwCDsQzJWEChjVu5sE>

https://www.jianguoyun.com/p/DXYHJ1wQzJWEChjWu5sE
